# Impact of the COVID-19 pandemic on type 2 diabetes care and factors associated with care disruption in Kenya and Tanzania

**DOI:** 10.1080/16549716.2024.2345970

**Published:** 2024-05-22

**Authors:** Richard E. Sanya, Caroline H. Karugu, Peter Binyaruka, Shukri F. Mohamed, Lyagamula Kisia, Peter Kibe, Irene Mashasi, Grace Mhalu, Christopher Bunn, Manuela Deidda, Frances S. Mair, Eleanor Grieve, Cindy M. Gray, Sally Mtenga, Gershim Asiki

**Affiliations:** a Chronic Diseases Management Unit, African Population and Health Research Center, Nairobi, Kenya; b Department of Health Systems, Impact Evaluation and Policy, Ifakara Health Institute, Dar es Salaam, Tanzania; c School of Social and Political Sciences, University of Glasgow, Glasgow, UK; d School of Health and Wellbeing, University of Glasgow, Glasgow, UK; e Department of Women’s and Children’s Health, Karolinska Institute, Stockholm, Sweden

**Keywords:** Diabetes, COVID-19, chronic disease care, health systems, Africa

## Abstract

**Background:**

The COVID-19 pandemic affected healthcare delivery globally, impacting care access and delivery of essential services.

**Objectives:**

We investigated the pandemic’s impact on care for patients with type 2 diabetes and factors associated with care disruption in Kenya and Tanzania.

**Methods:**

A cross-sectional study was conducted among adults diagnosed with diabetes pre-COVID-19. Data were collected in February–April 2022 reflecting experiences at two time-points, three months before and the three months most affected by the COVID-19 pandemic. A questionnaire captured data on blood glucose testing, changes in medication prescription and access, and healthcare provider access.

**Results:**

We recruited 1000 participants (500/country). Diabetes care was disrupted in both countries, with 34.8% and 32.8% of the participants reporting change in place and frequency of testing in Kenya, respectively. In Tanzania, 12.4% and 17.8% reported changes in location and frequency of glucose testing, respectively. The number of health facility visits declined, 14.4% (*p* < 0.001) in Kenya and 5.6% (*p* = 0.001) in Tanzania. In Kenya, there was a higher likelihood of severe care disruption among insured patients (adjusted odds ratio [aOR] 1.56, 95% confidence interval [CI][1.05–2.34]; *p* = 0.029) and a lower likelihood among patients residing in rural areas (aOR, 0.35[95%CI, 0.22–0.58]; *p* < 0.001). Tanzania had a lower likelihood of severe disruption among insured patients (aOR, 0.51[95%CI, 0.33–0.79]; *p* = 0.003) but higher likelihood among patients with low economic status (aOR, 1.81[95%CI, 1.14–2.88]; *p* = 0.011).

**Conclusions:**

COVID-19 disrupted diabetes care more in Kenya than Tanzania. Health systems and emergency preparedness should be strengthened to ensure continuity of service provision for patients with diabetes.

## Paper Context

- **Main findings:** The COVID-19 pandemic disrupted diabetes care in Kenya and Tanzania resulting in changes in place and frequency of blood glucose testing, medication prescribed (less oral hypoglycaemics and more insulin), fewer health facility visits and more difficulty accessing healthcare providers.
- **Added knowledge:** This study quantifies the impact of the COVID-19 pandemic on diabetes care in Kenya and Tanzania, and describes the factors associated with care disruption in both countries.
- **Global health impact for policy and action:** Evidence on diabetes care disruption is useful in making plans and policies responsive to the needs of diabetes patients during pandemics or related emergency situations.

## Background

The outbreak of coronavirus disease 2019 (COVID-19), caused by the severe acute respiratory syndrome coronavirus 2, resulted in a global pandemic [1]. The pandemic, responsible for an estimated 770 million confirmed infections and 7 million deaths as of 21 September 2023, affected healthcare delivery globally [2,3]. In Africa, 9.5 million infections were documented by the World Health Organization by 21 September 2023 [2]. At the peak of the pandemic, routine care for chronic diseases was adversely affected worldwide, with diabetes, chronic obstructive pulmonary disease, and hypertension most affected [4]. The World Health Organization introduced the COVID-19 *Strategic Preparedness and Action Plan* to provide public health support measures to respond to the pandemic and ensure continuity of essential services [5]. African countries faced significant challenges, due to the fragile health systems with inadequate healthcare resources and personnel [6,7]. The shift of healthcare resources to the COVID-19 emergency response and measures instituted by governments to control the spread of the virus, such as lockdown and ‘stay-at-home’ measures disrupted care [8,9].

An efficient and functional healthcare system results in effective diabetes care. Type 2 diabetes, a chronic disease, requires continuity of care involving routine consultation, blood glucose monitoring, screening and management of complications and co-morbidities. Discontinuity of care results in poor control and an increased risk of complications [10]. The COVID-19 pandemic did not spare patients with type 2 diabetes, with evidence showing an increase in the proportion of patients with poor treatment control in Ethiopia from 24.8% pre-COVID-19 to up to 36.% during the pandemic [11] and experts highlighting the importance of timely and effective management [12]. Also, having type 2 diabetes predisposes individuals to severe forms of COVID-19 [13]. However, evidence of the impact of the pandemic on diabetes care is limited in the two East African countries of Kenya and Tanzania. According to estimates by the International Diabetes Federation, in 2021, 821,500 (3% prevalence) and 2,884,000 (10.3% prevalence) adults had diabetes in Kenya and Tanzania, respectively [14]. In Kenya and Tanzania provide two interesting contexts for this assessment because of the quite different approaches deployed to manage the pandemic. Whereas Kenya instituted strict lockdowns and curfews at the peak of the pandemic, Tanzania opted for a less stringent approach without lockdowns and curfews. However, it is not known whether these two different approaches have led to different outcomes in terms of diabetes care in the two countries.

Knowledge of the impact of the COVID-19 pandemic on diabetes care is helpful in planning care for patients with type 2 diabetes and chronic diseases in pandemic situations. Such evidence will be essential to strengthen healthcare services and to make national plans and policies responsive to the needs of type 2 diabetes patients during pandemics or related emergency situations. Therefore, we investigated the extent of healthcare disruption resulting from the COVID-19 pandemic and associated factors among patients with type 2 diabetes in Kenya and Tanzania.

## Methods

### Study design and setting

This was a cross-sectional survey study conducted in urban and rural locations in Kenya and Tanzania in East Africa between February – April 2022. In Kenya surveys were conducted in four counties: Nairobi and Kiambu (predominantly urban); and Nyeri and Vihiga (rural). In Tanzania, surveys were conducted in Dar es Salaam (urban) and Morogoro (rural) regions.

### Participants

Patients with type 2 diabetes receiving care at selected health facilities were invited to participate. Lists of type 2 diabetes patients receiving care from these health facilities were obtained from patient records. We included consenting adults (aged ≥18 years) of all gender, with or without co-morbidities (such as hypertension), who had commenced type 2 diabetes care before the COVID-19 pandemic declaration in Kenya and Tanzania (March 2020). Patients who were bedridden, pregnant or breastfeeding and those unable to comprehend study information were excluded.

### Variables

The outcome variables measured were: (1) change in where testing took place; (2) change in frequency of blood glucose testing; (3) change in prescribed medication; (4) change in access to prescribed medication; (5) change in number of health facility visits in the preceding three months; (6) change in the cost of medication; and (7) change in access to a health provider. A ‘disruption’ variable was developed as an unweighted composite measure of the outcome variables and categorised into ‘no disruption’, ‘moderate disruption’, and ‘severe disruption’. Disruption was considered ‘severe’ if four or more of the above outcomes were experienced and ‘moderate’ if one to three outcomes were experienced. Socio-demographic and health-related variables, such as age, sex, location, marital status, occupation, education, economic status, health insurance coverage, family history of diabetes, presence of co-morbidities and known duration with type 2 diabetes were measured and considered as potential predictors of disruption. Socio-economic status was assessed subjectively using an economic ladder question (1–10 scale) because most of the participants did not have formal employment with regular income to estimate wealth and the ladder has been validated as a measure of socioeconomic status among such populations (graphically represented with 10 rungs) [15].

### Data sources/measurements

The study protocols and tools were aligned across countries to ensure consistency. Before the commencement of the surveys, the field teams were trained, and study tools piloted. In collaboration with healthcare providers, experienced, trained fieldworkers contacted study participants to make appointments. Informed consent was obtained from eligible participants and a questionnaire administered. With the questionnaire, data were collected on socio-demographic characteristics, medical history and the impact of the COVID-19 pandemic on diabetes care. Participants were asked to report on their experiences at two time-points, during the three months before COVID-19 was confirmed in both countries (March 2020) and the three months during the pandemic when they felt their care was most affected. Also, information was collected on reasons for any disruption of care. Interviews were conducted by telephone in Kenya and face-to-face in Tanzania, owing to the differing COVID-19 regulations in the two countries.

### Sample size considerations

We planned to recruit 500 participants in each country (1000 participants). The sample size was estimated using the Cochran formula [16], assuming 50% of patients with type 2 diabetes experienced disruption of care during COVID-19 [17], considering a power of 80% at 95% confidence interval, 5% acceptable sampling error and a non-response rate of 30%.

### Statistical analysis

All statistical analyses were done using STATA version 16 (College Station, Texas). Descriptive characteristics of individuals in Kenya were reported alongside those of Tanzania and presented as frequencies and percentages as well as mean and standard deviation for continuous variables. Differences in the individual disruption variables before and during the COVID-19 were assessed for statistically significant differences using the independent samples T-test. We described the levels of disruption (none, moderate and severe) graphically using bar charts. To identify predictors of disruption in three levels, we conducted a univariate ordered logistic regression and then, a multivariate ordered logit model including pre-selected co-variates was built. The selection of factors was made a priori and based on the WHO social determinants of health and wellbeing framework [18]. Crude and Adjusted odds ratios, 95% confidence intervals and p-values are reported. A p-value of less than 0.05 was considered statistically significant. The analyses are country-specific and presented alongside each other.

## Results

Between February and April 2022, 1000 participants (500 in each country) consented to participate in the study. In Kenya, most participants were recruited from Nairobi (276) and Kiambu (104) counties. In Tanzania, 300 and 200 participants were recruited from Dar es Salaam and Morogoro, respectively.

The characteristics of the study participants are shown in Table 1. The participants were predominantly female (66% [330/500] and 67.2% [336/500] in Kenya and Tanzania, respectively). In both countries, the participants were of similar age (Kenya 58.2 years [standard deviation (SD) 12.6] and Tanzania 56.8 years [SD 10.2]). However, in the above 70 category, Kenya had 18.2% while Tanzania had 6.2%. Most of the participants were of low socio-economic status (Kenya 89.4% (447/500), Tanzania 74.8% (374/500)). In Kenya, 55.2% of the participants had secondary education and higher while 33.6% had the same in Tanzania.

**Table 1.** Socio-demographic characteristics of type 2 diabetes patients who participated in the study in Kenya and Tanzania.

Variable		Kenya (*N*=500)	Tanzania (*N*=500)
		n (%)	n (%)
Location*	Urban	380 (76.0%)	300 (60.0%)
	Rural	120 (24.0%)	200 (40.0%)
Sex	Female	330 (66.0%)	336 (67.2%)
	Male	170 (34.0%)	164 (32.8%)
Age in years, Mean (SD)		58.2 (12.6)	56.8 (10.2)
Age group	<40 years	39 (7.8%)	36 (7.2%)
	40–49 years	80 (16.0%)	62 (12.4%)
	50–59 years	135 (27.0%)	165 (33.0%)
	60–69 years	155 (31.0%)	206 (41.2%)
	>70 years	91 (18.2%)	31 (6.2%)
Formal education	None	16 (3.2%)	34 (6.8%)
	Primary	208 (41.6%)	298 (59.6%)
	Secondary	201 (40.2%)	126 (25.2%)
	College/University	75 (15.0%)	42 (8.4%)
Occupation	Formal employment	31 (6.2%)	40 (8.0%)
	Farming (small and large scale)	78 (15.6%)	103 (20.6%)
	Self-employed (small and large business)	162 (32.4%)	190 (38.0%)
	Retired	55 (11.0%)	72 (14.4%)
	Unemployed/Homemaker	174 (34.8%)	95 (19.0%)
Family history of diabetes	No	233 (46.6%)	205 (41.0%)
	Yes	267 (53.4%)	295 (59.0%)
Duration from first diagnosis of type 2 diabetes	≥6 years	309 (61.8%)	334 (66.8%)
	<6 years	191 (38.2%)	166 (33.2%)
Health insurance coverage	Uninsured	163 (32.6%)	302 (60.4%)
	Insured	337 (67.4%)	198 (39.6%)
Presence of co-morbidities	Yes	355 (71.0%)	368 (73.6%)
	No	141 (29.0%)	132 (26.4%)
Economic status (ladder scale [1–10])	Lower SES** [1–5]	447 (89.4%)	374 (74.8%)
	Higher SES** [6–10]	53 (10.6%)	126 (25.2%)

*Kenya, Urban - Nairobi and Kiambu counties, Rural - Nyeri and Vihiga counties; Tanzania, Urban - Dar es Salaam, Rural – Morogoro.

**SES, Socio-economic status.

The proportions of participants who reported changes in diabetes care parameters due to the COVID-19 pandemic are shown in Table 2. Overall, a higher proportion of participants in Kenya compared to Tanzania reported changes across the parameters studied. In Kenya, blood glucose testing was the most disrupted component of care, with 34.8% (178/500) and 32.8% (164/500) changed place and frequency of testing in Kenya, respectively. In Tanzania, 12.4% (62/500) reported a change in place of testing and 17.8% (89/500) reported a change in the frequency of testing. In Kenya, there was a relative decline in testing at home (29.6% to 20%; p = 0.0019) and an increase in testing at health facilities (39.4–96.0%; p < 0.0001). In Tanzania, the reverse was observed with an increase in testing at home (2.6–7.2%; p < 0.001) and a decline in testing at health facilities (86.6–81.0%; p < 0.0001). Participants did less testing during the pandemic in Kenya as shown by a decline in testing at least once a day and an increase in testing at least twice a week. In both countries, there was a decline noted in the prescription of oral hypoglycaemics (Kenya, −2.6% [p < 0.0001]; Tanzania, −2.2% [p = 0.002]) and an increase noted for prescription of insulin (Kenya, 2.6% [p < 0.0019]; Tanzania, 2.2% [p = 0.002]) during the pandemic. There was a 14.4% (p < 0.0001) decline in the number of health facility visits in Kenya and a 5.6% (p = 0.001) decline in Tanzania.

**Table 2.** Diabetes care before and during the COVID-19 pandemic in Kenya and Tanzania.

		Kenya			Tanzania		
Variable		Pre-COVID-19 N (%)	During the pandemic N (%)	Change % (*p value)	Pre-COVID-19 N (%)	During the pandemic N (%)	Change % (*p value)
**Blood glucose testing**							
Where testing took place	Total number (%) who changed testing place		174 (34.8)			62 (12.4)	
	Tested at home	148 (29.6)	100 (20.0)	**-9.6 (0.002)**	13 (2.6)	36 (7.2)	**4.6 (<0.001)**
	Tested at health facility	197 (39.4)	381 (96.0)	**56.6 (<0.001)**	433 (86.6)	405 (81.0)	**-5.6 (<0.001)**
	Tested both at home and health facility	155 (31.0)	19 (3.8)	**-27.2 (<0.001)**	54 (10.8)	59 (11.8)	1.0 (0.197)
Frequency of testing	Total number (%) who changed frequency of testing		164 (32.8)			89 (17.8)	
	Tested at least once in a day	167 (33.4)	150 (30.0)	-3.4 (0.434)	45 (9.0)	52 (10.4)	1.4 (0.280)
	Tested once or twice a week	232 (46.4)	211 (42.2)	**-4.2 (<0.001)**	28 (5.6)	37 (7.4)	1.8 (0.129)
	Tested at least once a month	87 (17.4)	98 (19.6)	**2.2 (0.001)**	316 (63.2)	281 (56.2)	**-7.0 (<0.001)**
	Tested at least once in 3 months	8 (1.6)	19 (3.8)	1.6 (0.784)	62 (12.4)	76 (15.2)	**2.8% (0.031)**
**Medication prescription, access, and use**							
Medication Prescription	Number (%) who had a change of prescription		96 (19.20)			63 (12.6)	
	Prescribed insulin only	45 (9.0)	59 (11.8)	**2.8 (0.002)**	46 (9.2)	57 (11.4)	**2.2 (0.002)**
	Prescribed oral hypoglycaemics only	415 (83.0)	402 (80.4)	**-2.6 (<0.001)**	450 (90.0)	439 (87.8)	**-2.2 (0.002)**
	Both insulin and oral hypoglycaemics	40 (8.0)	39 (7.8)	**-0.2 (<0.001)**	4 (0.8)	4 (0.8)	0
Access to medication	Accessed prescribed medications	452 (93.2)	346 (69.2)	**24 (<0.001)**	482 (96.4)	467 (93.4)	**-3.0 (0.009)**
Use of prescribed medication	Used medications as prescribed	483 (96.6)	485 (97.0)	0.4 (0.670)	489 (97.8)	480 (96.0)	-1.8 (0.061)
**Access to health facilities**							
Health facility visits	Visited hospital in the last 3 months	445 (89.0)	373 (74.6)	**-14.4 (<0.001)**	456 (91.2)	428 (85.6)	**-5.6 (0.001)**
Care access	Successfully accessed healthcare provider	494 (98.8)	464 (92.8)	**-6 (<0.001)**	498 (99.6)	493 (98.6)	-1.0 (0.096)
	Accessed in-person consultation	488 (98.8)	447 (96.3)	**-2.5 (<0.001)**	498 (99.6)	491 (98.2)	**-1.4 (0.035)**

*p values obtained from comparison of variables using the independent samples T-test.

**Figure 1.**
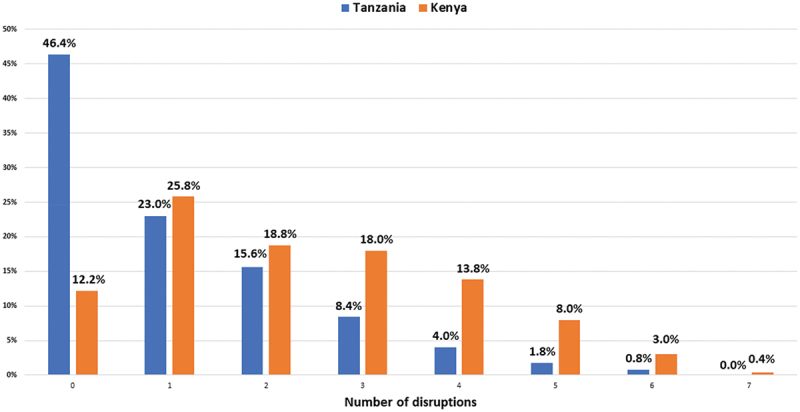
Proportion of cumulative disruptions of diabetes care disruption due to COVID-19 in Kenya and Tanzania.

**Figure 2.**
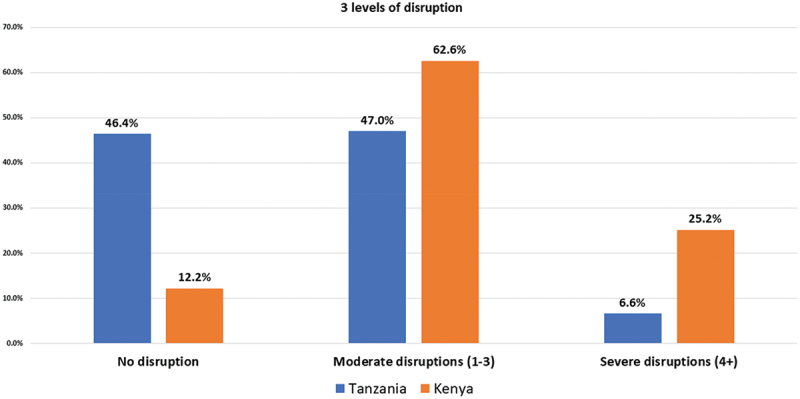
Levels of diabetes care disruption due to COVID-19 in Kenya and Tanzania.

A breakdown of the cumulative number of disruptions is shown in Figure 1. Of the participants who experienced disruption, the majority experienced moderate disruption (Kenya 62.6%, Tanzania 47%; Figure 2).

Disruptions assessed include change in where testing took place, frequency of testing, cost of testing, medication prescription access, cost of prescribed medication and access to health provider.

Disruptions assessed include change in where testing took place, frequency of testing, cost of testing, medication prescription access, cost of prescribed medication and access to health provider.

The factors associated with disruption are shown in Tables 3 and 4. In the bivariate analysis (Table 3), rural living was associated with less disruption in Kenya. In Tanzania, rural living, being older (70+ years) and having health insurance were associated with less disruption. However, being of low socioeconomic status was associated with severe disruption.

**Table 3.** Predictors of severe disruption of diabetes care in Kenya and Tanzania (bivariate analysis).

		Kenya		Tanzania	
Variable	Categories (n = Kenya, Tanzania)	Odds ratio (95%CI)	p value	Odds ratio (95%CI)	p value
Location	Urban (*n* = 380,300)				
	Rural (*n* = 120,200)	0.40 (0.26, 0.63)	<0.001	0.38 (0.26, 0.54)	<0.001
Sex	Female (330,336)				
	Male (*n* = 170, 164)	1.03 (0.71, 1.49)	0.875	0.88 (0.61, 1.27)	0.496
Age group	<40 (*n* = 39,36)				
	40–49 (*n* = 80,62)	1.56 (0.73, 3.34)	0.255	0.94 (0.41, 2.14)	0.888
	50–59 (*n* = 135,165)	0.95 (0.46, 1.93)	0.881	0.64 (0.31, 1.33)	0.231
	60–69 (*n* = 155,206)	0.71 (0.35, 1.45)	0.347	0.52 (0.26, 1.07)	0.078
	≥70 (*n* = 91,31)	0.84 (0.39, 1.79)	0.645	0.24 (0.09, 0.64)	0.005
Education Level	No formal education (*n* = 16,34)				
	Primary (*n* = 208, 298)	0.82 (0.29, 2.30)	0.710	0.73 (0.37, 1.45)	0.369
	Secondary (*n* = 201,126)	0.81 (0.29, 2.27)	0.691	0.50 (0.24, 1.04)	0.064
	College/University (*n* = 75,42)	0.74 (0.25, 2.20)	0.589	0.42 (0.17, 1.03)	0.058
Occupation	Formal employment (*n* = 31,40)				
	Farming (small and large scale) (*n* = 78,103)	0.89 (0.39, 2.03)	0.778	0.63 (0.31, 1.28)	0.201
	Self-employed (small and large business) (*n* = 162,190)	0.92 (0.43, 1.98)	0.828	1.62 (0.84, 3.12)	0.151
	Retired (*n* = 55,72)	0.58 (0.15, 2.24)	0.432	0.75 (0.36, 1.59)	0.457
	Unemployed (*n* = 174,92)	0.92 (0.43, 1.98)	0.828	1.43 (0.70, 2.92)	0.322
Family history of diabetes	No (*n* = 233,205)				
	Yes (*n* = 267,295)	1.03 (0.72, 1.47)	0.867	0.87 (0.62, 1.23)	0.433
Duration from first diagnosis of type 2 diabetes	≥6 years (*n* = 309,334)				
	<6 years (*n* = 191,166)	0.76 (0.52, 1.09)	0.136	1.22 (0.85, 1.75)	0.286
Health insurance coverage	Uninsured (*n* = 163,302)				
	Insured (*n* = 337,198)	1.16 (0.80, 1.69)	0.425	0.35 (0.24, 0.50)	<0.001
Presence of co-morbidities	No (*n* = 141,132)				
	Yes (*n* = 355,368)	0.97 (0.93, 1.02)	0.209	1.00 (0.83, 1.19)	0.965
Economic status (ladder scale 1–10)	Higher SES [6–10] (*n* = 126,126)				
	Lower SES [1–5] (*n* = 374,374)	0.64 (0.36, 1.13)	0.127	2.58 (1.71, 3.88)	<0.001

**Table 4.** Predictors of severe disruption of diabetes care in Kenya and Tanzania (multivariate analysis).

		Kenya		Tanzania	
Variable	Categories (n=Kenya, Tanzania)	Odds ratio* (95%CI)	p value*	Odds ratio* (95%CI)	p value*
Location	Urban (*n*=380,300)				
	Rural (*n*=120,200)	**0.35 (0.22, 0.58)**	**<0.001**	0.72 (0.45, 1.15)	0.169
Sex	Female (330,336)				
	Male (*n*=170, 164)	1.08 (0.70, 1.67)	0.717	1.08 (0.69,1.68)	0.699
Age group	<40 (*n*=39,36)				
	40–49 (*n*=80,62)	1.57 (0.71, 3.47)	0.266	0.81 (0.34, 1.94)	0.638
	50–59 (*n*=135,165)	0.94 (0.44, 1.98)	0.866	0.61 (0.27, 1.34)	0.216
	60–69 (*n*=155,206)	0.66 (0.31, 1.40)	0.279	0.59 (0.26, 1.34)	0.210
	≥70 (*n*=91,31)	0.86 (0.38, 1.95)	0.719	0.37 (0.12, 1.17)	0.092
Education Level	No formal education (*n*=16,34)				
	Primary (*n*=208, 298)	0.68 (0.23, 1.99)	0.487	0.71 (0.34, 1.48)	0.357
	Secondary (*n*=201,126)	0.66 (0.22, 1.95)	0.454	0.63 (0.27, 1.49)	0.294
	College/University (*n*=75,42)	0.54 (0.17, 1.72)	0.294	0.48 (0.17, 1.36)	0.165
Occupation	Formal employment (*n*=31,40)				
	Farming (small and large scale) (*n*=78,103)	1.10 (0.44, 2.79)	0.835	0.48 (0.21, 1.09)	0.080
	Self-employed (small and large business) (*n*=162,190)	0.76 (0.33, 1.75)	0.515	0.77 (0.36, 1.65)	0.499
	Retired (*n*=55,72)	0.54 (0.13, 2.20)	0.394	0.81 (0.35, 1.90)	0.630
	Unemployed (*n*=174,92)	0.88 (0.37, 2.06)	0.763	0.70 (0.30, 1.68)	0.428
Family history of diabetes	No (*n*=233,205)				
	Yes (*n*=267,295)	1.10 (0.76, 1.58)	0.619	0.97 (0.67, 1.42)	0.881
Duration from first diagnosis of type 2 diabetes	≥6 years (*n*=309,334)				
	<6 years (*n*=191,166)	0.72 (0.49, 1.06)	0.093	0.93 (0.63, 1.38)	0.731
Health insurance coverage	Uninsured (*n*=163,302)				
	Insured (*n*=337,198)	**1.56 (1.05, 2.34)**	**0.029**	**0.51 (0.33, 0.79)**	**0.003**
Presence of co-morbidities	No (*n*=141,132)				
	Yes (*n*=355,368)	0.97 (0.93, 1.02)	0.301	1.08 (0.89, 1.33)	0.431
Economic status (ladder scale 1–10)	Higher SES [6–10] (*n*=126,126)				
	Lower SES [1–5] (*n*=374,374)	0.79 (0.43, 1.43)	0.434	**1.81 (1.14, 2.88)**	**0.011**

*Adjusted for patient demographic and clinical characteristics (sex, age, education level, occupation, location, family history and duration of diabetes, presence of co-morbidities, economic status and health insurance coverage).

In the multivariate analysis (Table 4), the results are as follows. In Kenya, there was a higher likelihood of severe disruption among insured participants compared to the uninsured (adjusted odds ratio [aOR] 1.56 95% confidence interval [CI] [1.05, 2.34] p = 0.029). There was a lower likelihood of severe disruption among participants residing in rural areas (aOR 0.35[0.22–0.58] p < 0.001). A weak association with less disruption was noted for participants who have lived with type 2 diabetes for less than six years (aOR 0.72[0.49–1.06] p = 0.093). In contrast, the factors associated with severe disruption in Tanzania were health insurance cover and economic status. Participants with health insurance were less likely to be severely disrupted (aOR 0.51[0.33, 0.79] p = 0.003). However, participants from the lower socio-economic stratum were more likely to be severely disrupted (aOR 1.81[1.14, 2.88] p = 0.011). Some borderline association (lower likelihood) with severe disruption was observed for older participants (70+ years) (aOR 0.37[0.12, 1.17] p = 0.092) and farmers (aOR 0.48[0.21, 1.09] p = 0.080).

## Discussion

In this paper, we have shown that the COVID-19 pandemic disrupted diabetes care in Kenya and Tanzania. The disruption resulted in changes in place and frequency of blood glucose testing, medication prescribed (less oral hypoglycaemics and more insulin), fewer health facility visits and more difficulty accessing healthcare providers. Health insurance was a predictor of severe disruption in both countries but in different directions. In Kenya, having health insurance was positively associated with severe disruption, whereas there was an inverse association in Tanzania. Urban living and belonging to a lower economic status were associated with severe disruption in Kenya and Tanzania, respectively.

The disruption of diabetes care highlighted here has been reported elsewhere. For example, the pandemic resulted in an increase in poor control of diabetes and hypertension in Ethiopia [11]. Patients with diabetes in Rwanda reported challenges with care access [19]. Significant reductions were noted in outpatient visits and glycated haemoglobin (HbA1c) measurements among U.S. veterans in the first three months of the pandemic [20] and in diabetic patients in Italy [21]. There was disruption of care and a reduction in clinic visits by patients with diabetes in India [22].

Diabetes care was disrupted in both Kenya and Tanzania despite the two countries deploying different approaches to combat the pandemic. Patients with type 2 diabetes are at a higher risk of developing severe forms of COVID-19 and death due to COVID-19 compared to individuals without co-morbidities [13]. Patients were well informed about this vulnerability during the pandemic and fear of exposure to COVID-19 could potentially have deterred visits to healthcare facilities [4]. Furthermore, travel restrictions in Kenya and loss of employment and income due to the pandemic in both countries may have impacted diabetes care seeking.

Disruption of care was not only limited to diabetes. The pandemic resulted in the reduction of general inpatient utilization in Kenya [23]. More broadly, there was a reduction in service utilization of maternal, newborn and child health services in eastern Africa [9,24] and disruption of cancer, TB and HIV services [3,25,26]. The pandemic also affected planned healthcare delivery and resource allocation for persons with chronic diseases [27].

Our study reported a decline in prescriptions of oral hypoglycaemics and an increase in insulin prescriptions among patients with type 2 diabetes. The disruption of care may have resulted in poor blood sugar control, necessitating a prescription change. Also, the poor control could have resulted from limited access to healthy and affordable food, limited physical activity opportunities due to restrictions on movement, and increased stress. The pandemic increased stress and anxiety for many people, which can affect blood sugar control [28].

Urban living was associated with severe disruption in Kenya. The pandemic impacted livelihoods and may have affected the urban areas more than the rural ones. Many people living in urban areas work in the informal sector and may have lost their livelihoods due to the pandemic. For example, lockdowns disrupted businesses and employment in Nairobi [29], making it difficult for people to afford healthcare. Also, lockdown measures enforced in Kenya (including the ban of public transport) may have impacted urban and rural areas differently, considering that public transportation is a major mode of travel in urban areas, which impedes travel to healthcare facilities. Furthermore, we cannot rule out differences in baseline care in rural and urban areas. With less formalised and poorer baseline care, there could have been less disruption in rural areas compared to urban areas. In addition, fewer COVID-19 infections were reported in rural areas and there was the common belief that COVID-19 was an ‘urban’ disease. This may have resulted in lower risk perception of the disease in rural areas [30].

Belonging to a lower economic status was associated with severe disruption in Tanzania. Low economic status can lead to more disruption of diabetes care during the COVID-19 pandemic by limiting access to healthcare facilities, medication, testing supplies, information, and education. People with low socio-economic status may live in areas with limited access to healthcare facilities, making it difficult to receive regular diabetes care. This problem can be exacerbated during the pandemic when movement restrictions and limited transport services make it difficult for people to travel to healthcare facilities. Also, there are challenges with affording medication and testing supplies for diabetes care. During the pandemic, these challenges were exacerbated by supply chain disruptions and economic hardships [31,32].

Having health insurance is expected to result in better healthcare access and outcomes [33]. Health insurance can help individuals access diabetes care during the pandemic by reducing the financial burden of healthcare expenses. It was, therefore, surprising to find that health insurance was a predictor of severe diabetes care disruption in Kenya. Health insurance has been shown to have limited effects on quality of care in low-income countries [34]. In some situations, health insurance could potentially disrupt diabetes care during the pandemic. Some health insurance plans in Kenya may not provide adequate coverage for diabetes services [35]. Finally, health insurance may not effectively ensure access to diabetes care if healthcare systems are overwhelmed with COVID-19 patients. In such situations, even individuals with health insurance may face challenges accessing diabetes care due to diverted resources and limited healthcare capacity.

Our study had several strengths. Our data are from two countries that adopted quite different approaches to the pandemic, providing two different contexts to diabetes care disruption. Also, the study design was robust and the sample size adequate, ensuring that the results are valid, reliable and generalizable. The study had some limitations, which we acknowledge. The data collection was cross-sectional, leading to temporal ambiguity and also making it difficult to infer causation. This was mitigated by collecting data for two time points, before and during the pandemic’s peak. Because of the retrospective nature of data, we cannot rule out recall bias. We also acknowledge that conditions in Kenya and Tanzania are not the same, influenced by the different country contexts. Lastly, the selection of the study sites was purposive and not random. To ensure that nationally representative data were collected, urban and rural study sites were selected in both countries.

The disruption of diabetes care during the COVID-19 pandemic in Kenya and Tanzania highlights several important issues. Our findings demonstrate the need for strong healthcare infrastructure support to ensure continuity of care during pandemic or related emergency situations for people with type 2 diabetes to ensure access to regular check-ups, and medication. Both countries should learn from the disruptions in healthcare caused by the pandemic and invest in improving healthcare infrastructure, including increasing the number of healthcare workers and expanding access to healthcare facilities. Telemedicine had emerged as a vital tool during the pandemic, allowing healthcare providers to provide remote care and consultation to patients with diabetes. Some telemedicine initiatives have been deployed in Kenya [36–38] and Tanzania [39]. Investment in expanding access to telemedicine services for people with diabetes would be beneficial [12,40].

## Conclusions

The pandemic has highlighted the importance of social safety nets and ensuring continuity of essential health services to protect vulnerable populations, including people with diabetes who may have lost their livelihoods due to the pandemic. Investing in social safety nets ensures that people with diabetes have access to affordable medication and testing supplies, even during economic hardship. Overall, the disruption of diabetes care during the COVID-19 pandemic in Kenya and Tanzania highlights the need for stronger healthcare infrastructure, more innovative approaches to diabetes care, and social safety nets to protect vulnerable populations. By learning from these disruptions, Kenya and Tanzania can work towards improving diabetes care and mitigating the impact of future pandemics on diabetes care.

## Data Availability

The datasets used and/or analyzed during the current study are available from the corresponding author on reasonable request.
